# Validating hip- and wrist-ActiGraph accelerometer cut-points for physical activity intensities in people living with coronary heart disease

**DOI:** 10.1371/journal.pone.0349618

**Published:** 2026-05-28

**Authors:** Nicole Freene, Brad Clark, Maria Bäck, Theo Niyonsenga, Kate Pumpa, Arjun Rangaraj, Tze Hao Wong, Soraya Joseph, Ahmed Khan, Rachel Davey, Amanda Lönn

**Affiliations:** 1 Health Research Institute, University of Canberra, Canberra, Australia; 2 Research Institute for Sport and Exercise, University of Canberra, Canberra, Australia; 3 Department of Health, Medicine and Caring Sciences, Unit of Physiotherapy, Linköping University, Linköping, Sweden; 4 Department of Occupational Therapy and Physiotherapy, Sahlgrenska University Hospital, Gothenburg, Sweden; 5 Institute for Sport and Health, University College Dublin, Dublin, Ireland; 6 Canberra Health Services, Canberra, Australia; 7 Department of Physical Activity and Health, The Swedish School of Sport and Health Sciences, Gymnastik- Och Idrottshögskolan (GIH), Lidingövägen 1, Stockholm, Sweden; Kurume University School of Medicine, JAPAN

## Abstract

People with coronary heart disease (CHD) are encouraged to meet the public health moderate-to-vigorous aerobic physical activity (MVPA) guidelines for secondary prevention of cardiovascular disease. However, no accelerometer aerobic intensity cut-points are currently available to classify MVPA in this population. This study aimed to establish absolute and relative aerobic physical activity intensity accelerometer cut-points in people with CHD and compare the new with existing cut-points in an international cohort. Eighty-six participants with CHD performed a resting-metabolic-rate (RMR) assessment, activities-of-daily-living (ADLs) and a peak treadmill test with mixed-chamber gas analysis while wearing two ActiGraph GT3X accelerometers (hip and wrist). The average RMR was 2.8 ml.kg^-1^.min^-1^, 20% less than the commonly used 1 Metabolic Equivalent of Task (3.5 ml.kg^-1^.min^-1^). The study sample was randomly split into a training and independent validation set (2:1) allowing for cross validation. In the training set, there were significant positive correlations between accelerometer counts.min^-1^ (y-axis, vector-magnitude (VM)) and intensity (relative and absolute) across both accelerometer hip- and wrist-placements for all activities (p < 0.001). Using Generalized Estimating Equation modelling, there was a strong linear relationship between accelerometer counts and absolute intensity for hip-placement (R^2^ = 0.62–0.71), and weaker relationships for hip relative intensity (R^2^ = 0.40–0.47) and wrist-placement (R^2^ = 0.09–0.25). In the validation set, Bland-Altman plots found that the mean differences between predicted and actual absolute and relative intensity measures were negligible for all accelerometer counts.min^-1^ (y-axis, VM) and placements (hip, wrist), although the dispersion of the differences (95% limits of agreement) were wide. Hip VM counts.min^-1^ cut-points were found to best identify absolute and relative MVPA. In the international comparison (n = 176), participants completed significantly more MVPA using the new cut-points (p < 0.001). Thus, accelerometer cut-points developed in healthy individuals appear to under-estimate physical activity intensity in this population and cut-points specific to people with CHD should be used.

Australian New Zealand Clinical Trials Registry: ACTRN12623000605695.

## Introduction

One in three myocardial infarctions are repeat events [1]. Not only are repeat myocardial infarctions more likely to be fatal, they are costly [1]. People with coronary heart disease (CHD) are encouraged to meet the public health physical activity guidelines to prevent repeat events and premature death [2]. To determine whether guidelines are being met, accurate measurement of physical activity is essential. Accelerometry measures of physical activity have been found to be more reliable and valid compared with self-report measures [3,4]. However, despite advances in technology, categorisation of physical activity intensity using accelerometry still exist [5].

Currently, accelerometer cut-point equations used to categorise physical activity intensity in people with CHD have been based on studies with healthy adults [5]. Accurately measuring physical activity in clinical and older sub-groups is a challenge as some movements may be slow and difficult to capture. Additionally, small changes in physical activity may lead to important health effects in these groups [3]. An absolute intensity approach may also not be the most accurate when examining physical activity in different disease states and age groups, as maximal oxygen uptake and resting metabolic rate decreases with age and clinical conditions, such as CHD [6,7].

Accelerometer measured physical activity is reported as low within cardiac rehabilitation, with only 15% of participants with CHD meeting the physical activity guidelines [4,8]. It is currently unclear if cardiac rehabilitation participants with CHD are not meeting the physical activity guidelines or being inaccurately classified. Accelerometer anatomical placement may also impact accelerometer cut-point thresholds [9]. Therefore, our research question is, does the use of accelerometer cut-points developed in healthy individuals underestimate the intensity of physical activity in people with CHD? Specifically, the aims of this study were to: (i) establish ActiGraph GT3X cut-points for hip (right) and wrist (non-dominant) wear to differentiate between absolute (metabolic equivalents, METs) and relative (percentage peak oxygen uptake, %VO_2_peak) physical activity intensities (light, moderate, vigorous) in people with CHD; and (ii) compare the new and existing cut-points in an international cohort of cardiac rehabilitation participants with CHD to determine if there were differences in daily moderate-to-vigorous physical activity (MVPA).

## Methods

### Study design

The validation study was conducted in Australia between August 2023 and December 2024. Ethics approval was received from the University of Canberra Human Research Ethics Committee (HREC-1872). All participants provided written informed consent. The trial was registered prospectively on the Australian New Zealand Clinical Trials Registry (ACTRN12623000605695).

### Participants

Using voluntary sampling, community-dwelling participants were recruited by advertising the study at cardiology clinics and phase II cardiac rehabilitation programs in Australia. Participants were included if they were aged ≥18 years old, had stable CHD and were receiving optimal medical treatment + /- revascularisation. Participants were excluded if they had: a primary diagnosis of atrial fibrillation; New York Heart Association class III-IV symptoms of heart failure; uncontrolled arrhythmias; severe chronic obstructive pulmonary disease; uncontrolled hypertension; symptomatic peripheral artery disease; unstable angina; uncontrolled diabetes; and were unable to perform a maximal walking test on a treadmill. Participants needed to have adequate English and cognitive skills to provide informed consent and follow instructions for the tests. Medical clearance screening was undertaken by the participant’s medical practitioner prior to inclusion and by a research team medical practitioner on the day of testing. All participants provided informed written consent. Characteristics of participants such as socio-demographic variables (i.e., gender, age, education level) and clinical predictor variables (i.e., time since cardiac event/procedure, cardiac-related medication, other medical conditions) were collected to describe the sample.

### Outcome measures

All outcome measures were collected on the same day in a University Sports Laboratory. On the test day, participants took their usual medications, refrained from eating for three hours before the test, and avoided exercise and caffeine. Participants arrived by taxi, were familiarised with the laboratory and received verbal instructions about the testing procedure. A resting-metabolic-rate (RMR) assessment was completed first, followed by activities of daily living (ADL) and a peak incremental treadmill test to minimise fatigue.

#### Anthropometric characteristics.

Height (cm), weight (kg) and body mass index (BMI, kg.m^-2^) were recorded once using a calibrated set of SECA scales and stadiometer (GmbH & Co., Hamburg, Germany).

#### Accelerometry.

Participants were asked to wear ActiGraph GT3X triaxial accelerometers over their right hip and on the dorsal aspect of their non-dominant wrist throughout the testing. All accelerometers were initialized at 30 Hz and data was downloaded in 1-second epochs and counts.min^-1^ for the y-axis (vertical axis; uniaxial) and vector magnitude (VM; triaxial). Vector magnitude is a composite measure of all three accelerometer axes (√x^2^ + y^2^ + z^2^)). To record timing of activities, a clock was used to synchronise the accelerometer clock and oxygen uptake measures.

#### Oxygen uptake.

Oxygen uptake (VO_2;_ ml.kg^-1^.min^-1^) was measured using a stationary mixed-chamber gas exchange analysis system (TrueOne, Parvomedics, Sandy, UT, USA) during all testing. Heart rate and blood pressure were monitored throughout using a 12-lead electrocardiogram (ECG; Norav Medical Inc. Delray Beach, FL, USA) and blood pressure cuff.

### Resting metabolic rate assessment

For the RMR assessment, participants were supine in a quiet room for 25-minutes. The average VO_2_ from the 10-to-20th minutes was used to record RMR. Extreme values were removed.

### Activities of daily living

Participants completed four ADL simulations: (1) watching television while sitting in a chair; (2) standing trunk stretching by twisting the sternum side to side every 30 seconds; (3) floor sweeping; and (4) stepping-in-place to a pace of 60 beats.min^-1^ [10]. Each ADL was completed for five-minutes according to standardized instructions [11].

### Peak treadmill test

The bespoke ramped treadmill protocol comprised of four five-minute stages at 3, 4, 5, and 6 km.h^-1^. For stage five, the speed remained constant (6 km.h^-1^) but the gradient increased by 3.5% every minute until volitional exhaustion or test termination by the research team. Peak VO_2_ was calculated as the highest 30-second average during the ramped treadmill test. Respiratory exchange ratio (RER) >1.1 [12–14] and ECG heart rate within 10% of age-predicted heart rate maximum (heart rate maximum (bpm)=220-age) [15] were recorded to determine if participants reached VO_2_max.

### Sample size

Using the general guideline for regression sample size (50 + 8*number of predictors) and four predictor variables (counts.min^-1^, gender, age, BMI), the sample size needed was 82 participants to explore the relationship of accelerometer counts with absolute intensity (METs) and relative intensity (%VO_2_peak) [16].

### Secondary analysis of international accelerometry data

Between 2015−2018, a cross-sectional study was conducted at two outpatient cardiac rehabilitation centres in Australia and Sweden [17]. Participants were commencing cardiac rehabilitation following a percutaneous coronary intervention (PCI; Australia n = 49, Sweden n = 127). A triaxial accelerometer (ActiGraph ActiSleep or GT3X) was used to assess physical activity in both countries. Participants were asked to wear the monitor on their right hip for seven-consecutive days. All data was sampled and downloaded as raw data (30 Hz) and converted to 15-second epochs (time interval) and counts.min^-1^ using the ActiLife software.The Sasaki VM 3 cut-points were used to determine time spent in MVPA (≥2690 counts.min^-1^). These processing methods when using the Sasaki cut-points have been used in prior research in this population [18–21], however, they have not been validated in people with CHD. The international data was re-processed and converted to 1-s epochs and counts.min^-1^.The new VM hip worn cut-points were applied to allow a comparison between the new and Sasaki cut-points using the same data capture methods (i.e.,: placement, triaxial) but different processing methods (i.e.,: epoch length, cut-points) as recommended. The characteristics of participants in the international cohort have been described elsewhere [17].

### Data analysis

The last minute of each stage of the treadmill test and ADLs was used to reflect counts.min^-1^ and VO_2_. Absolute intensity (METs) was calculated by dividing VO_2_ by RMR (ml.kg^-1^.min^-1^). Relative intensity was calculated as a percentage of peak VO_2_. Accelerometer counts across all activities were summarised in the total sample and subgroups (age groups, gender, BMI groups (above and below median)) using box plots.

The study sample was randomly split into a training and independent validation set (2:1), allowing for a cross-validation procedure. The normality of the variables was assessed by visualization, the Shapiro-Wilk test, and by examining skewness and kurtosis. In the training set, to further examine differences in counts.min^-1^ in sub-groups across all activities, the Chi-square test and Wilcoxon test for categorical variables was used. To explore the correlation between accelerometer counts (y-axis, VM) with absolute and relative intensity, repeated measures correlation was used [22]. The correlation was interpreted as weak (r < 0.10), modest (r = 0.1–0.29), moderate (r = 0.3–0.49), strong (r = 0.5–0.79), or very strong (r = 0.8–1.0) [23]. To explore the association of accelerometer counts (y-axis, VM) with absolute and relative intensity, unadjusted and adjusted linear generalized estimating equation (GEE) models were used, with participant id as a unique identifier (R library geepack). The adjusted models used the covariates age, gender and BMI as they are known physical activity correlates [24] Counts.min^-1^ cut-points were calculated by rearranging regression equations to solve for upper and lower margins of light, moderate and vigorous absolute (METs) and relative (%VO_2_peak) intensity [14]. A sensitivity analysis using GEE was also completed excluding counts.min^-1^ > 5000 on the y axis and >8000 counts.min^-1^ for VM after visual inspection of the scatterplots.

Using the validation set, Bland-Altman plots were used to determine differences between the actual and predicted intensity (absolute and relative) and the average of the two measures. Mean differences (bias), 95% confidence interval for the bias and 95% limits of agreement (LoA) were calculated [25]. Linear regression was used to check whether the mean difference (MD) varied across average values of actual and predicted intensity after visual examination of the plots [26].

Mean time spent in daily minutes of MVPA for the two new cut-points (absolute and relative intensity) were compared to commonly used accelerometer cut-points (Sasaki VM cut-points [21]) using paired Wilcoxon signed rank sum tests in the international cardiac rehabilitation accelerometer data (Australia and Sweden, n = 176). All analyses were conducted using R Studio version 2023.03.1. or SPSS version 27. Significance level was set at p < 0.05.

## Results

Eighty-six participants completed all testing (Table 1). Most participants were male, self-reported they were diagnosed with CHD six-years ago and their most recent cardiac procedure had been a PCI. No participants were smokers, and few had type 2 diabetes, but most had other chronic diseases and were on blood pressure and cholesterol medications. There were no differences between the training and validation cohorts except the validation cohort were more recently diagnosed with CHD. Most participants requested to stop the treadmill test. At the end of the treadmill test 52% reached a heart rate ≥90% of the predicted heart rate max; 33% reached RER ≥ 1.10; and 29% reached both criteria. The average RMR was 2.8 ml.kg^-1^.min^-1^, 20% less than the commonly used 1 MET value of 3.5 ml.kg^-1^.min^-1^.

**Table 1:** Descriptive characteristics of participants.

Characteristic	Training (n = 60)	Validation (n = 26)	Total (n = 86)
Age *(yr)*, mean (SD)	66 (10)	67 (11)	66 (10)
Gender, number male (%)	50 (83)	25 (96)	75 (87)
Country of birth, number Australia (%)	37 (62)	19 (73)	56 (65)
Relationship status, number partner (%)	50 (8)	21 (81)	71 (83)
Tertiary education, number (%)	38 (63)	16 (62)	54 (63)
Employment status, number not in labour force (%)	37 (62)	16 (62)	53 (62)
Days from first CHD diagnosis, median (IQR)	2,496 (6,005)	1,521 (1,985)	2,201 (5,137)
Days from most recent cardiac event, median (IQR)	1,290 (1,936)	1,062 (1,080)	1,221 (1,719)
Most recent cardiac event/procedure			
Coronary artery bypass graft, number (%)	11 (18)	5 (19)	16 (19)
Percutaneous coronary intervention, number (%)	42 (70)	20 (77)	62 (72)
Myocardial infarction	7 (12)	1 (3.8)	8 (9)
Current smokers, number yes (%)	0 (0)	0 (0)	0 (0)
Type 2 diabetes, number yes (%)	7 (12)	0 (0)	7 (8.1)
No other chronic diseases, number (%)	40 (67)	17 (65)	57 (66)
Blood pressure medication, number yes (%)	47 (78)	22 (85)	69 (80)
Betablockers, number yes (%)	22 (39)	7 (28)	29 (35)
Cholesterol medication, number yes (%)	58 (97)	26 (100)	84 (98)
Other cardiac medications, number yes (%)	51 (85)	22 (85)	73 (85)
Body mass index *(kg.m*^*-2*^), mean (SD)	27.4 (3.7)	27.5 (4.5)	27.5 (4.0)
Resting metabolic rate *(ml.min*^*-1*^*.kg*^*-1*^), mean (SD)	2.78 (0.48)	2.97 (0.43)	2.84 (0.47)
VO_2_peak *(ml.min*^*-1*^*.kg*^*-1*^), mean (SD)	23 (8)	25 (9)	24 (8)

CHD, coronary heart disease; MVPA, Moderate-to-Vigorous Physical Activity; VO_2_, oxygen uptake.

### Establishing absolute and relative intensity cut-points

**Fig 1:**
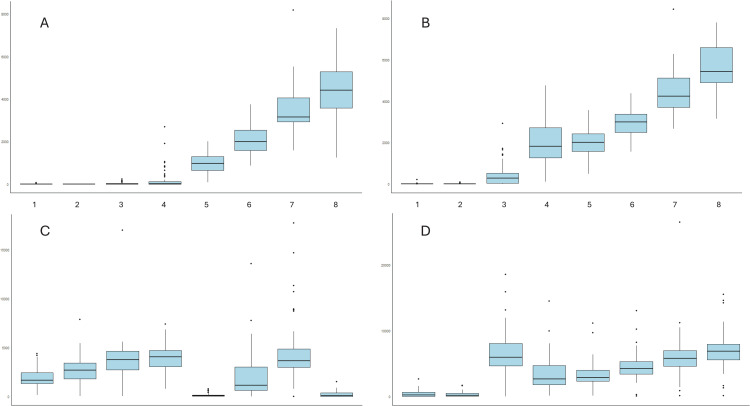
Box plot of accelerometer counts.min^-1^ across different activities for different accelerometer counts and placement. **(A) y-axis, hip; (B) vector magnitude (VM), hip; (C) y-axis, wrist; (D) VM, wrist.** The solid line in the in the middle of each box represents the median. The box represents the interquartile range (Q1-Q3). The lines outside the box (whiskers) show the smallest and largest range within 1.5 times the IQR from Q1 and Q3, respectively. Activities: (1) watching television while sitting; (2) standing trunk stretching; (3) floor sweeping; (4) stepping in place; (5) treadmill stage 1; (6) treadmill stage 2; (7) treadmill stage 3; (8) treadmill stage 4.

For accelerometer hip placement (Fig 1A & 1B) an increase in uniaxial (y-axis) and triaxial (VM) counts.min^-1^ with increasing intensity of activity was found. This relationship was more variable for wrist placement (Fig 1C & 1D). Results generally found no difference in counts.min^-1^ across age groups, gender and BMI groups for all activities (Supplementary Figs 1–3 in S1 File). However, there was significant difference in counts.min^-1^ (y-axis, VM) for hip placement between BMI groups for all treadmill stages (p < 0.05). In repeated measures correlations, there were significant positive correlations between accelerometer counts and intensity (relative and absolute) across both placements for all activities (Table 2). The correlations were very strong for hip placement, compared to moderate-strong for wrist placement.

**Table 2:** Repeated correlation between accelerometer counts and oxygen uptake in the training set (n = 60).

Accelerometer counts	Percentage VO_2_peak	METs
Hip		
Y-axis, *r (p)*	0.82 (<0.001)	0.85 (<0.001)
Vector magnitude, *r (p)*	0.87 (<0.001)	0.90 (<0.001)
Wrist		
Y-axis, *r (p)*	0.39 (<0.001)	0.43 (<0.001)
Vector magnitude, *r (p)*	0.55 (<0.001)	0.56 (<0.001)

VO_2_, oxygen uptake; MET, metabolic equivalent.

There was a strong linear relationship between accelerometer counts and absolute intensity for hip placement in the unadjusted GEE model (R^2^ = 0.62–0.71; Table 3). The strength of the relationship was weaker for relative intensity with hip placement (R^2^ = 0.40–0.47). The weakest relationship was between accelerometer counts and intensity (absolute and relative) for wrist placement (R^2^ = 0.09–0.25). The inclusion of covariates (age, gender, BMI; Supplementary table 1) or a sensitivity analysis (Supplementary table 2) did not improve the model fit.

**Table 3:** New absolute (METs) and relative (%VO_2_peak) physical activity intensity accelerometer prediction equations.

Accelerometer counts	Prediction equation^a^			
	Intercept	Slope	R^2^	SEE
Absolute intensity				
Hip				
Y-axis	2.284	0.001	0.619	1.042
VM	1.947	0.001	0.709	0.916
Wrist				
Y-axis	2.915	0.0001	0.086	1.59
VM	2.672	0.0001	0.251	0.52
Relative intensity				
Hip				
Y-axis	31.8	0.007	0.397	17.1
VM	26.3	0.01	0.474	17.1
Wrist				
Y-axis	39.6	0.001	0.086	21.1
VM	37.8	0.001	0.176	20.5

VM, vector magnitude; SEE, standard error of the estimate.

^a^Unadjusted generalised estimating equations.

**Fig 2:**
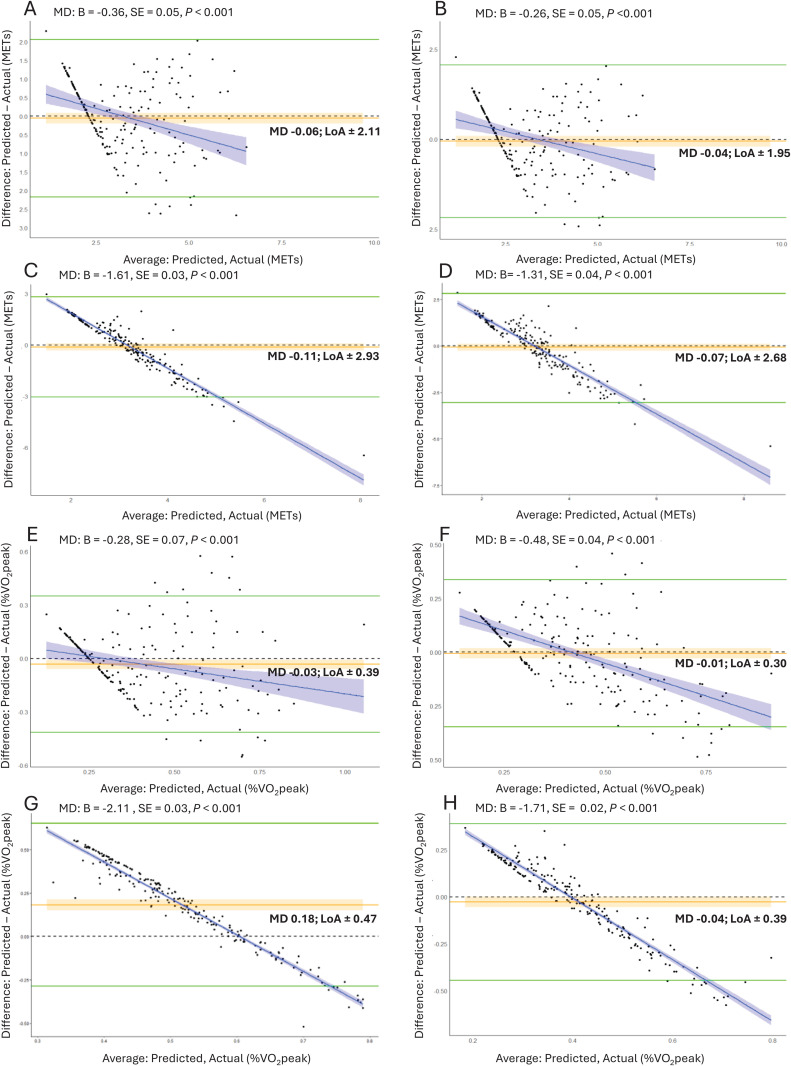
Bland-Altman plots for average predicted and actual absolute (METs) and relative intensity (%VO_2_peak) for different accelerometer counts and placement. **(A) METs, y-axis, hip; (B) METs, vector magnitude (VM), hip; (C) METs, y-axis, wrist; (D) METs, VM, wrist; (E) %VO2peak, y-axis, hip; (F) %VO2peak, VM, hip; (G) %VO2peak, y-axis, wrist; (H) %VO2peak, VM, wrist.** MD, crude mean difference; LoA, crude limits of agreement; MD: B, slope of the regression line; SE, standardised error for the regression line. Orange line = crude mean difference (bias); Orange shading = 95% confidence interval for the bias; Green line = crude LoA; Dotted black line = zero difference; Purple line = regression line; Purple shading = 95% confidence interval for the regression line. Participants are represented by solid dots.

The crude mean difference between predicted and actual absolute and relative intensity measures was negligible for all accelerometer counts (y-axis, VM) and placements (hip, wrist; Fig 2). However, the dispersion of the differences (crude 95% LoA) was wide for all accelerometer counts and placements (Fig 2). Linear regression analysis showed a significant negative association for all accelerometer counts and placements for the difference between predicted and actual absolute and relative intensity and the average of the two measures. Both absolute and relative intensity tended to be over-predicted at lower intensities and under-predicted at higher intensities. The slope of the regression line was less for hip placement compared to the wrist indicating hip worn accelerometry may provide a more accurate prediction of intensity.

### International comparison

Considering the results from the GEE and Bland-Altman plots, VM counts.min^-1^ and hip placement were found to best identify absolute and relative MVPA in people with CHD. The newly proposed cut-points were determined by rearranging the GEE equation and using the ACSM light, moderate and vigorous absolute and relative ranges [14] (Table 4). In the international sample there were significant differences between the new and Sasaki cut-points for daily MVPA with the largest difference seen between the absolute and Sasaki cut-points (Table 5). The new absolute moderate intensity cut-point was lower than the Sasaki cut-point resulting in a significant difference in daily MVPA (130 vs 57 mins.day^-1^; p < 0.001). The new relative moderate intensity cut-point was higher than Sasaki cut-point, while the vigorous intensity cut-point was lower, resulting in a significant difference in daily MVPA (80 vs 57 mins.day^-1^; p < 0.001). The new cut-points showed the same patterns of physical activity across countries.

**Table 4:** New absolute (METs) and relative (%VO_2_peak) physical activity intensity accelerometer cut-points.

Accelerometer VM counts	Cut-points		
	Light	Moderate	Vigorous
Absolute intensity^^a^^,^^b^^			
Hip	82-1630	1631-6278	≥6279
Relative intensity^^a^^,^^c^^			
Hip	1514-2782	2783-5319	≥5320
Sasaki^b^			
Hip	–	2690-6166	≥6167

VM, vector magnitude.

^a^Developed using 1-second epochs.

^b^Sedentary/very light: < 2 metabolic equivalents (METs), light: 2–2.9 METs, moderate: 3–5.9 METs, vigorous: ≥ 6 METs.

^c^Sedentary/very light: < 37% VO_2_peak, light: 37–45% VO_2_peak, moderate: 46–63% VO_2_peak, vigorous: ≥ 64% VO_2_peak.

**Table 5:** International comparison of daily moderate-to-vigorous physical activity using the new and existing accelerometer cut-points.

MVPA mins/day^a^	Australia (n = 49)	Sweden (n = 127)	Total (n = 176)
Absolute intensity (1s epoch)	110.10 (35.09)	137.29 (38.83)	129.72 (39.65)
Relative intensity (1s epoch)	62.78 (28.46)	86.66 (31.24)	80.01 (32.25)
Sasaki (15s epoch)	42.65 (26.81)	62.17 (28.61)	56.73 (29.38)

^a^Data presented as mean (standard deviation); MVPA, moderate-to-vigorous physical activity.

## Discussion

ActiGraph GT3X accelerometer cut-points for hip and wrist wear to differentiate between absolute (METs) and relative (%VO2peak) physical activity intensities in people with CHD have been developed. Our findings suggest that hip triaxial (VM) cut-points provide the most appropriate method for identifying absolute and relative MVPA in people with CHD. In the international cohort, the newly developed hip triaxial cut-points found higher levels of MVPA compared to existing cut-points commonly used in this population. Thus, the use of accelerometer hip triaxial cut-points specific to people with CHD is recommended for improved measurement of physical activity.

Only one other pilot study has explored uniaxial and triaxial ActiGraph GT3X accelerometer relative intensity cut-points in people with CHD [27]. The inclusion criteria for participants were similar to our study, although participants were younger and had higher levels of cardiorespiratory fitness. Participants completed a ramp treadmill protocol only with gas analysis to calculate relative intensity. Accelerometer placement was not clear. The final triaxial moderate intensity cut-point (1800 counts.min^-1^) was similar to our hip absolute but much lower than our relative intensity cut-point. Similarly, inclusion of age, gender and BMI did not improve the model fit, as has been reported in other studies [9,28]. Absolute triaxial hip MVPA cut-points developed in older people (≥60 yrs) are also higher than those found in our study (2857 counts.min^-1^ [9]; 1924 counts.min^-1^ [28]; 2751 counts.min^-1^ [29]). This may be explained due to compromised cardiorespiratory function from CHD [2] lowering cardiorespiratory fitness at the same age, the use of different methods to develop the cut-points and using the standard 1 MET (3.5 ml.kg^-1^.min^-1^) as the RMR to calculate METs.

Despite negligible differences between predicted and actual absolute and relative intensity measures for all accelerometer counts and placements, the dispersion of the differences were wide and widest for wrist accelerometry. This indicates the estimates were poor at the individual level, but the predictive equations are useful to determine intensity at a group level. This has been reported in other studies in older adults, finding similar crude mean differences and limits of agreement for triaxial hip accelerometry [29]. The poorer performance of wrist-worn accelerometry is likely due to increased upper limb movement during lower intensity activities (i.e., trunk rotation, sweeping, stepping in place), resulting in acceleration and oxygen uptake not increasing proportionally. This may indicate that wrist accelerometers have difficulty classifying physical activity intensity when individuals undertake a variety of activities across the energy expenditure continuum throughout the day [30].

Comparing the two newly developed hip worn triaxial cut-points to the commonly used Sasaki cut-points [21] in this population [17] we found participants completed significantly more MVPA. When using the new absolute intensity cut-points, daily MVPA minutes were over double compared to daily MVPA minutes using the Sasaki cut-points. The Sasaki cut-points were developed in 32 healthy participants with a mean age of 28 (SD 9) years and a BMI of 23.8 (SD 3.6) using 1-second epochs and absolute intensity [21]. In our CHD cohort, participants were much older, with a mean age of 66 (SD 10) years. Additionally, to calculate absolute intensity (METs) Sasaki used the standard 1 MET (3.5 ml.kg^-1^.min^-1^) as the RMR. In our study we measured individual RMR and our mean RMR was much lower at 2.8 ml.kg^-1^.min^-1^. This means that calculated METs (VO_2_/RMR) would be much higher for any given VO_2_ in our cohort. This lower RMR is supported by the literature with the same average RMR reported in a study involving 70 people with CHD [31] and older adults (≥60 yrs) [6,28]. The Mark cut-points were also compared to the Sasaki cut-points in 230 people with CHD who wore an ActiGraph GT3X accelerometer in free-living conditions [32]. Participants were similar in age, BMI and fitness to our cohort. In alignment with our findings, participants completed nearly double the amount of daily MVPA using the Mark compared to the Sasaki cut-points. Thus, clinicians and researchers should be cautious when choosing ActiGraph cut-points to apply to accelerometer data in people with CHD.

### Strengths and limitations

This appears to be the first study to develop absolute and relative intensity cut-points in people with CHD using uniaxial and triaxial accelerometer acceleration and two anatomical positions. A major strength of this study is using individual RMR measurements. Additionally, the use of ADLs and a ramp treadmill test allowed the intensity of activities across the energy expenditure continuum to be explored. The use of a stationary gas analysis system to measure oxygen uptake is also generally more valid and reliable compared to portable gas analysis systems [33]; although does not allow gas analysis in free-living conditions. Other limitations include that only half of the participants reached at least one of the criteria for a maximal effort treadmill test, potentially limiting the validation of vigorous intensity cut-points. The participants were also predominantly male, obese, tertiary educated and free of other chronic disease. This limits the generalisability of the results to other people with CHD, such as females and broader age groups.

## Conclusions

ActiGraph accelerometer cut-points developed in healthy individuals appear to under-estimate the intensity of physical activity in people with CHD. Additionally, hip triaxial accelerometry appears to more accurately capture physical activity intensity in this cohort across a variety of physical activities compared to wrist accelerometers at a group level.

## Supporting information

S1 File Supplementary figures and tables file. (DOCX)
