# Supplementary material for: Validating hip- and wrist-ActiGraph accelerometer cut-points for physical activity intensities in people living with coronary heart disease
Source: PLoS One. 2026 May 28;21(5):e0349618. doi: 10.1371/journal.pone.0349618 (PMC13218503; doi:10.1371/journal.pone.0349618)
Supplement: S1 File — (DOCX) [file pone.0349618.s001.docx]

**Supplementary Files**

Supplementary Figure 1. Box plot of accelerometer counts per minute across different activities for different accelerometer counts and placement by age subgroups. (A) y-axis, hip; (B) vector magnitude (VM), hip; (C) y-axis, wrist; (D) VM, wrist. The solid line in the in the middle of each box represents the median. The box represents the interquartile range (Q1-Q3). The lines outside the box (whiskers) show the smallest and largest range within 1.5 times the IQR from Q1 and Q3, respectively.

(A)


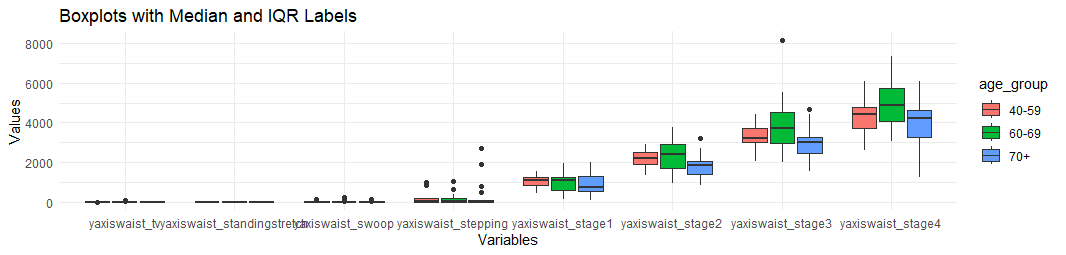


(B)


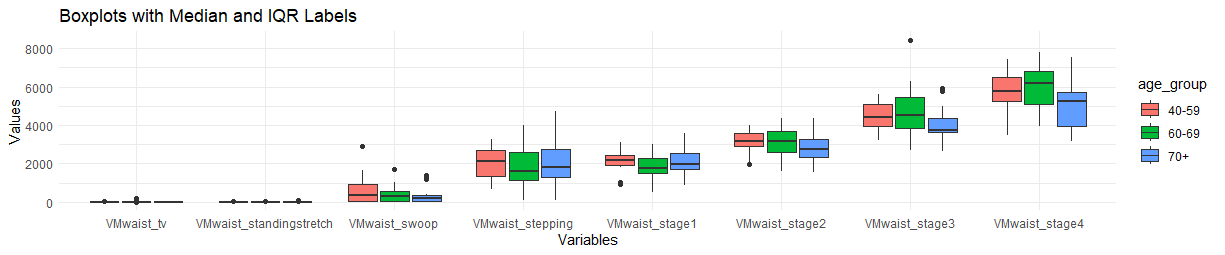


(C)


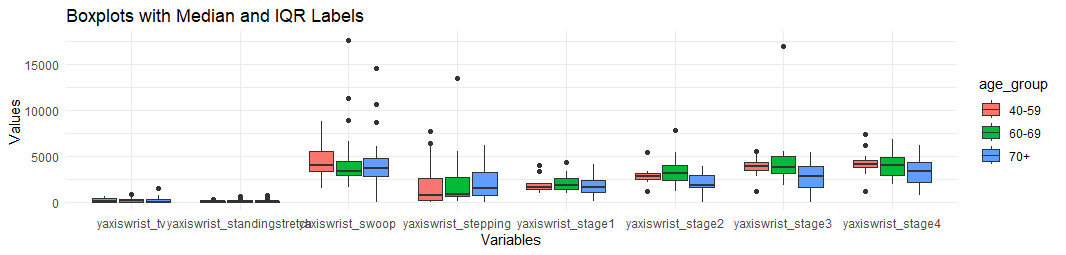


(D)


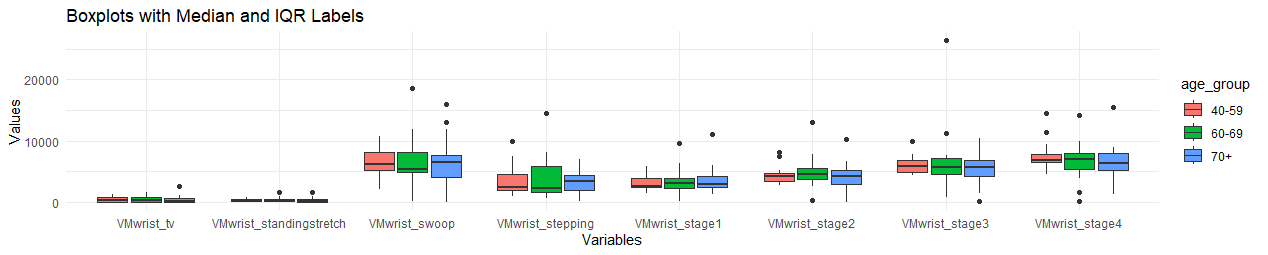


Supplementary Figure 2. Box plot of accelerometer counts per minute across different activities for different accelerometer counts and placement by gender. (A) y-axis, hip; (B) vector magnitude (VM), hip; (C) y-axis, wrist; (D) VM, wrist. The solid line in the in the middle of each box represents the median. The box represents the interquartile range (Q1-Q3). The lines outside the box (whiskers) show the smallest and largest range within 1.5 times the IQR from Q1 and Q3, respectively.

(A)


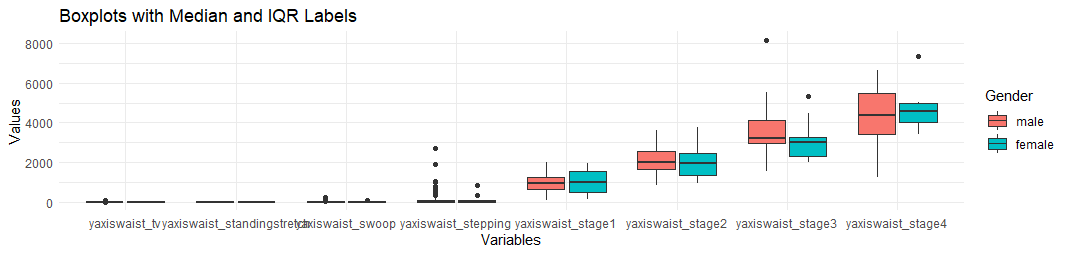


(B)


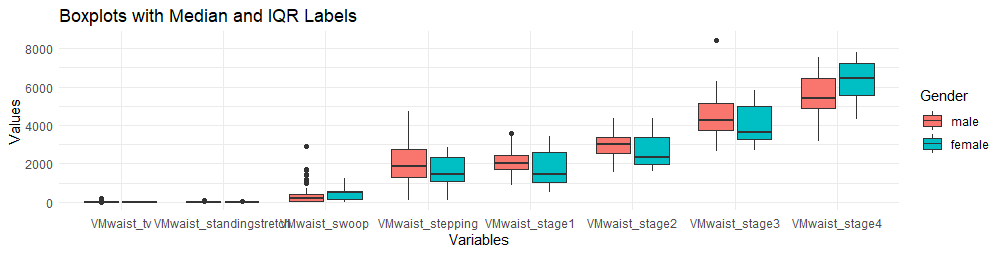


(C)


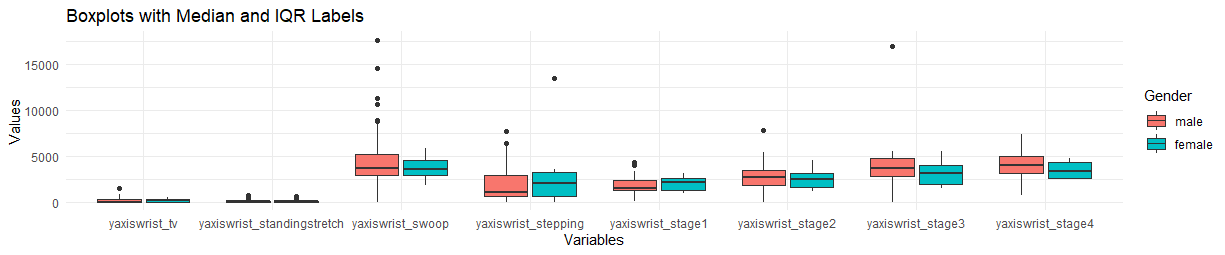


(D)


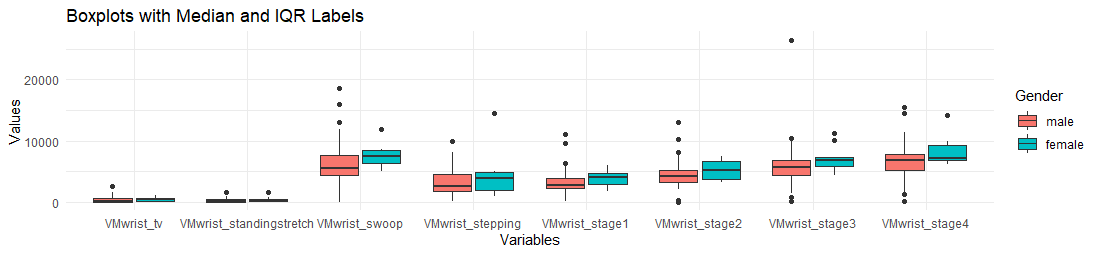


Supplementary Figure 3. Box plot of accelerometer counts per minute across different activities for different accelerometer counts and placement by body mass index (BMI) sub-groups. (A) y-axis, hip; (B) vector magnitude (VM), hip; (C) y-axis, wrist; (D) VM, wrist. The solid line in the in the middle of each box represents the median. The box represents the interquartile range (Q1-Q3). The lines outside the box (whiskers) show the smallest and largest range within 1.5 times the IQR from Q1 and Q3, respectively.

(A)


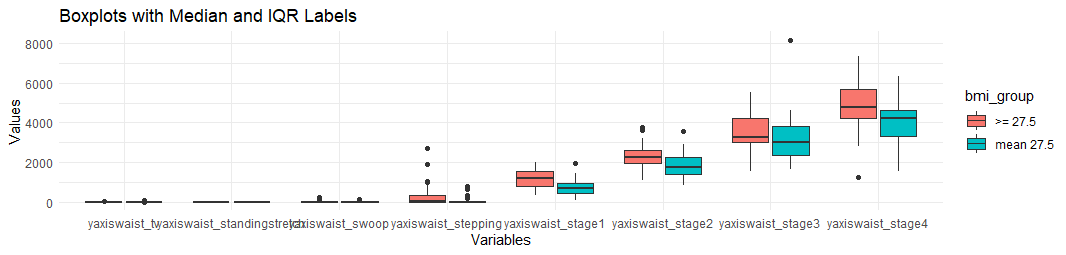


(B)


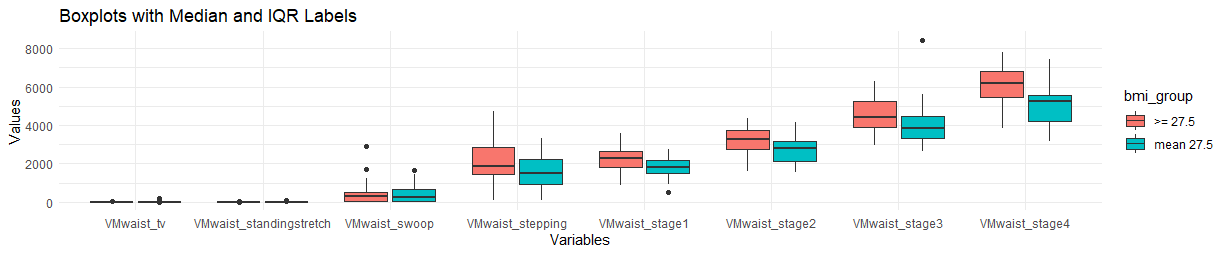


(C)


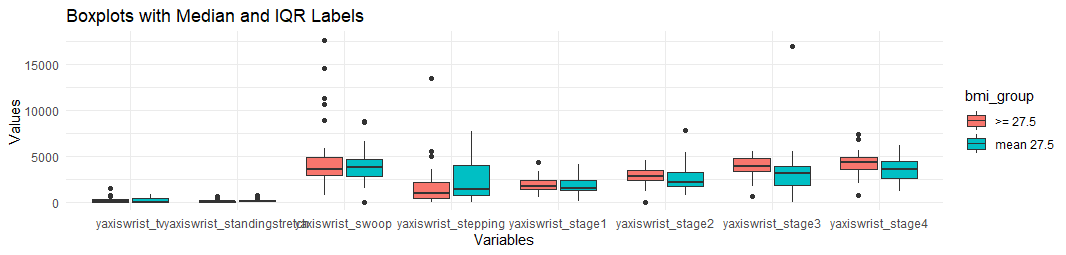


(D)


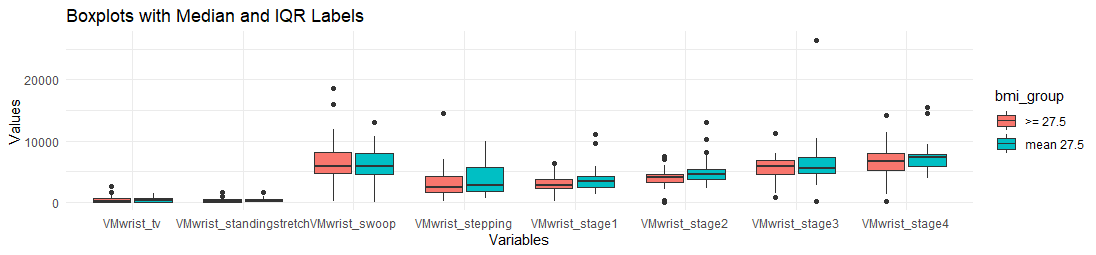


Supplementary Table 1. Newly developed absolute (METs) and relative (%VO_2_peak) physical activity intensity accelerometer prediction equations (adjusted).

| Accelerometer counts | | | Prediction equation^a^ | | | |
| --- | --- | --- | --- | --- | --- | --- |
|  |  |  | Intercept | Slope | R^2^ | SEE |
| Absolute intensity^b^ | | |  |  |  |  |
|  | Hip | |  |  |  |  |
|  | | Y-axis | 0.619 | 0.001 | 0.630 | 1.03 |
|  | | VM | 0.711 | 0.001 | 0.719 | 0.902 |
|  | Wrist | |  |  |  |  |
|  | | Y-axis | 1.524 | 0.0001 | 0.134 | 1.58 |
|  | | VM | 1.078 | 0.0001 | 0.173 | 0.198 |
| Relative intensity^c^ | | |  |  |  |  |
|  | Hip | |  |  |  |  |
|  | | Y-axis | -21.7 | 0.001 | 0.493 | 15.7 |
|  | | VM | -22.8 | 0.01 | 0.576 | 14.2 |
|  | Wrist | |  |  |  |  |
|  | | Y-axis | -3.8 | 0.001 | 0.106 | 20.6 |
|  | | VM | -6.2 | 0.001 | 0.176 | 20.5 |

VM, vector magnitude; SEE, standard error of the estimate

^a^ Adjusted generalised estimating equations (age, gender, BMI)

Supplementary Table 2. Newly developed absolute (METs) and relative (%VO_2_peak) physical activity intensity accelerometer prediction equations (sensitivity analysis).

| Accelerometer counts | | | Prediction equation^a^ | | | |
| --- | --- | --- | --- | --- | --- | --- |
|  |  |  | Intercept | Slope | R^2^ | SEE |
| Absolute intensity | | |  |  |  |  |
|  | Hip | |  |  |  |  |
|  | | Y-axis | 2.296 | 0.001 | 0.594 | 1.040 |
|  | | VM | 1.955 | 0.001 | 0.704 | 0.898 |
|  | Wrist | |  |  |  |  |
|  | | Y-axis | 3.044 | 0.0005 | 0.345 | 1.31 |
|  | | VM | 2.224 | 0.0002 | 0.324 | 1.37 |
| Relative intensity | | |  |  |  |  |
|  | Hip | |  |  |  |  |
|  | | Y-axis | 49.3 | 0.002 | 0.401 | 19.4 |
|  | | VM | 30.2 | 0.001 | 0.467 | 19.8 |
|  | Wrist | |  |  |  |  |
|  | | Y-axis | 8.0 | 0.008 | 0.193 | 19.7 |
|  | | VM | 45.7 | 0.002 | 0.220 | 19.6 |

VM, vector magnitude; SEE, standard error of the estimate

^a^ Unadjusted generalised estimating equations
